# Open Reduction and Internal Fixation with Plate and Screw versus Triplanar External Fixation in the Surgical Treatment of Calcaneal Fractures: A Retrospective Cohort Study

**DOI:** 10.3390/jcm13133770

**Published:** 2024-06-27

**Authors:** Vito Pavone, Marco Sapienza, Michela Carnazza, Marco Simone Vaccalluzzo, Giulia Leotta, Francesco Sergi, Giuseppe Mobilia, Danilo Di Via, Gianluca Testa

**Affiliations:** Department of General Surgery and Medical Surgical Specialties, Section of Orthopaedics and Traumatology, University Hospital Policlinico “Rodolico-San Marco”, University of Catania, 95123 Catania, Italy; vitopavone@hotmail.com (V.P.); marcosapienza09@yahoo.it (M.S.); michela.carnazza@libero.it (M.C.); marcovaccalluzzo@hotmail.it (M.S.V.); giulialeotta94@gmail.com (G.L.); fsrgbl33@gmail.com (F.S.); mobiliagiuseppe87@gmail.com (G.M.); danilodivia91@gmail.com (D.D.V.)

**Keywords:** calcaneal fractures, external fixator, MIOS technique, plate and screw, retrospective cohort study

## Abstract

**Background**: The treatment of displaced intra-articular calcaneal fractures (DIACF) is debated. This study compares open reduction and internal fixation (ORIF) with minimally invasive osteosynthesis (MIOS). **Methods**: We conducted a retrospective study on 70 patients with DIACF treated between January 2018 and September 2022, divided into ORIF (*n* = 50) and MIOS (*n* = 20) groups. Functional outcomes were assessed using the Maryland Foot Score (MFS) and the Creighton-Nebraska Health Foundation Assessment Scale (CNHFAS). Radiographic outcomes, complication rates, and reintervention rates were evaluated. A chi-square analysis examined the correlation between Sanders classification and treatment choice. **Results**: The chi-square analysis indicated no significant correlation between the complexity of the fracture and the type of treatment chosen (χ^2^ = 0.175, *p* = 0.916). Additionally, the Cochran–Armitage test for trend showed no significant trend in the choice of treatment based on fracture complexity (statistic = 0.048, *p* = 0.826). A Kaplan–Meier analysis showed a longer time to reintervention for MIOS (*p* = 0.029). Complication rates were similar, with specific complications varying between groups. Quality-of-life outcomes were comparable. **Conclusions**: ORIF is preferable for high-demand patients due to better anatomical outcomes, while MIOS suits high-risk patients by reducing reinterventions and complications. Further randomized trials are needed to confirm these findings.

## 1. Introduction

Calcaneal fractures, which account for 2% of all bodily fractures and 60% of tarsal fractures, are frequently caused by high axial energy trauma. Of these, 75% are intra-articular fractures, which greatly influence postoperative results [1]. Displaced intra-articular calcaneus fractures (DIACF) typically stem from high-energy trauma, and optimal surgical outcomes necessitate a deep understanding of calcaneal anatomy, fracture characteristics, and associated soft tissue injuries [2]. How best to treat DIACF remains a contentious issue. The two main treatment options are open reduction and internal fixation (ORIF), where injuries are reduced with plates and screws, and minimally invasive osteosynthesis (MIOS), which uses percutaneous reduction and external fixation. The choice depends on the patient’s particulars such as age, functional expectations, existing health issues, degree of injury, and convalescence factors [3]. More surgeons are now leaning towards minimally invasive methods due to fewer soft tissue complications and improved postoperative recovery [2]. Nevertheless, these methods might result in inadequate anatomical reduction and inaccurate restoration of the Böhler’s angle, which is crucial for the functional recovery of the heel [4,5].

The Sanders classification is a widely used system to assess the severity of calcaneal fractures, guiding treatment decisions based on the complexity of the fracture. This classification divides intra-articular calcaneal fractures into four types based on the number and location of fracture lines in the posterior facet of the calcaneus. The classification plays a crucial role in determining the most appropriate surgical approach, with more complex fractures (Sanders types III and IV) often requiring more invasive techniques such as ORIF, while less severe fractures (Sanders type II) may be effectively managed with minimally invasive methods like MIOS. Historical treatment options for calcaneal fractures have evolved significantly, as outlined in recent studies [6]. Additionally, primary subtalar arthrodesis is another surgical option for highly comminuted Sanders IV intra-articular fractures, providing stability and reducing the risk of post-traumatic arthritis [7].

This study aimed to compare the outcomes of ORIF and MIOS in treating DIACF, focusing on patient satisfaction, complication rates, and overall treatment efficacy. By analyzing these factors, we sought to provide practical insights for clinical decision-making.

## 2. Materials and Methods

### 2.1. Study Design and Data Collection

This comparative retrospective cohort study involved 70 patients who underwent surgery for displaced intra-articular calcaneal fractures (DIACF) at the Orthopedics and Traumatology Unit of A.O.U. Policlinico “G. Rodolico” University of Catania, Italy, between January 2018 and September 2022. Data were obtained from medical records and supplemented by telephone interviews. This study followed the STROBE guidelines.

### 2.2. Demographics

Demographic data, including age, gender, weight, height, and comorbidities, were collected from medical records. Information on risk factors and health status prior to the trauma, such as the presence of decompensated diabetes, diabetic foot, hypertension, smoking, autoimmune diseases, immunodeficiencies, coagulopathies, peripheral arteriopathies, and other pathologies affecting performance status, was also gathered.

### 2.3. Inclusion and Exclusion Criteria

The inclusion criteria included patients over 18 years of age who were hospitalized between 1 January 2018 and 30 September 2022. These patients had intra-articular calcaneal fractures treated by specific methods and may or may not have had bone grafts. Polytraumatized patients were also included. The exclusion criteria included a history of trauma or foot surgery, open or beak fractures, fractures outside the joint, Sanders type I fractures, and cases treated with primary arthrodesis.

### 2.4. Fracture Patterns

Fracture patterns were categorized according to the Sanders classification, based on radiographic and CT scan assessments. This classification included Sanders type II (22 cases), type III (41 cases), and type IV (7 cases).

### 2.5. Radiographic Assessment

Radiographic outcomes were evaluated using the Böhler’s and Gissane’s angles, measured preoperatively and postoperatively. Radiographic evaluations were performed at 1, 3, 6, and 12 months postoperatively to monitor the progress of fracture healing and anatomical restoration.

### 2.6. Complication

Complications were classified as immediate, early, and late postoperative complications. Immediate complications included traumatic or hemorrhagic shock and vascular and nerve injuries. Early complications included thromboembolism, dehiscence and/or infection of surgical wounds, delays in wound healing time, with or without vacuum-assisted closure (VAC) therapy, compartment syndrome, and skin necrosis. Late complications included pneumonia, bedsores, chronic pain and swelling, tendinitis, joint stiffness, arthritis, algodystrophy resistant to painkillers, pseudoarthrosis, and consolidation defects [8,9].

### 2.7. Quality-of-Life Assessment

Quality of life was assessed using the Maryland Foot Score (MFS) and the Creighton-Nebraska Health Foundation Assessment Scale (CNHFAS) [1]. These scales evaluate various aspects of a patient’s performance status following a heel fracture, including the presence of chronic or sporadic pain, motor function, joint function, need for support devices, aesthetic deformities, and work recovery.

### 2.8. Surgical Techniques

The conventional approach of internal fixation with plate and screws (ORIF) and minimally invasive osteosynthesis with a triplane external fixator (MIOS) are described in detail.

For ORIF, patients were positioned on their side with a thigh tourniquet. An “L”-shaped incision was created, extending vertically between the fibula and Achilles tendon and horizontally at the base of the fifth metatarsal. A full-thickness flap was then lifted, ensuring the preservation of vascularization and the sural nerve. The peroneal retinaculum was split, and both the fibulo-calcaneal and talo-calcaneal ligaments were removed to expose the fracture site. The posterior tuberosity fragment was aligned with the medial fragment using an osteotome and temporarily secured with K-wires. Radiographic confirmation ensured precise reduction before internal fixation with screws and a plate. The plate used was typically a low-profile locking plate with multiple holes for screw fixation, allowing for angular stability. Screws varied in length from 20 mm to 40 mm, depending on the fracture configuration and bone quality. In a few cases, a synthetic bone graft was used to rebuild the depressed joint surface. Postoperatively, the ankle was immobilized with a knee-high pinstripe cast for a month; partial weight-bearing was allowed after 6 weeks and full weight-bearing after 8 weeks [5].

For MIOS, patients were positioned in lateral decubitus opposite the fracture site under loco-regional anesthesia. The lateral aspect of the heel was exposed, allowing for full lateral and axial fluoroscopic visualization. Two K-wires were inserted through the heel to achieve reduction, confirmed with an image intensifier. An external fixator was then applied, composed of three clamps and six grippers fitted with self-drilling bone screws. The screws used were typically 4 mm in diameter and 70–90 mm in length, providing adequate stability for fragment fixation. The fixator ensured the restoration of the bone fragments and the Böhler’s angle. Postoperatively, the patient was barred from weight-bearing for 4 weeks but allowed immediate active and passive ankle movements. The external fixator was removed after 6 weeks, with follow-up X-rays confirming successful recovery [10].

Photos of the surgical techniques are available upon request.

### 2.9. Follow-Up

Patients were followed up at regular intervals postoperatively (1, 3, 6, and 12 months) to monitor healing progress and any complications. Follow-up evaluations included clinical assessments, radiographic imaging, and quality-of-life measurements. This regular monitoring allowed for the early detection and management of any postoperative issues.

### 2.10. Endpoints

The primary endpoints were functional outcomes assessed using the MFS and CNHFAS scales. Secondary endpoints included radiographic outcomes (Böhler’s and Gissane’s angles) and the incidence of postoperative complications.

### 2.11. Statistical Analysis

Statistical analyses were conducted using SPSS software version 25.0. The Shapiro–Wilk test was used to examine the normal distribution of functional outcome scores. Independent sample *t*-tests compared means between the two groups, and chi-square tests assessed differences in categorical outcomes. A *p*-value below 0.05 indicated statistical significance in all tests. Continuous variables are presented as mean ± standard deviation, and categorical variables are presented as frequencies (percentages).

## 3. Results

### 3.1. Patient Demographics and Baseline Characteristics

A total of 70 patients were included in the study, with 50 patients undergoing ORIF and 20 patients receiving MIOS. The mean age of the patients was 46.6 ± 12.4 years in the ORIF group and 53.5 ± 11.2 years in the MIOS group (*p* = 0.032). The mean BMI was 25.8 ± 3.4 kg/m^2^ for the ORIF group and 26.3 ± 3.7 kg/m^2^ for the MIOS group (*p* = 0.452). The gender distribution was 28 males and 12 females in the ORIF group, and 14 males and 6 females in the MIOS group (*p* = 0.801). The mean waiting time for surgery was 9 ± 3 days for ORIF and 3 ± 2 days for MIOS (*p* < 0.001).

### 3.2. Fracture Classification

The fractures were classified according to the Sanders classification: 22 cases were type II, 41 cases were type III, and 7 cases were type IV. In the ORIF group, there were 15 type II, 30 type III, and 5 type IV fractures. In the MIOS group, there were 7 type II, 11 type III, and 2 type IV fractures. The distribution of fracture types did not significantly differ between the ORIF and MIOS groups (χ^2^ = 0.175, *p* = 0.916).

### 3.3. Correlation between Sanders Classification and Treatment Choice

**Figure 1:**
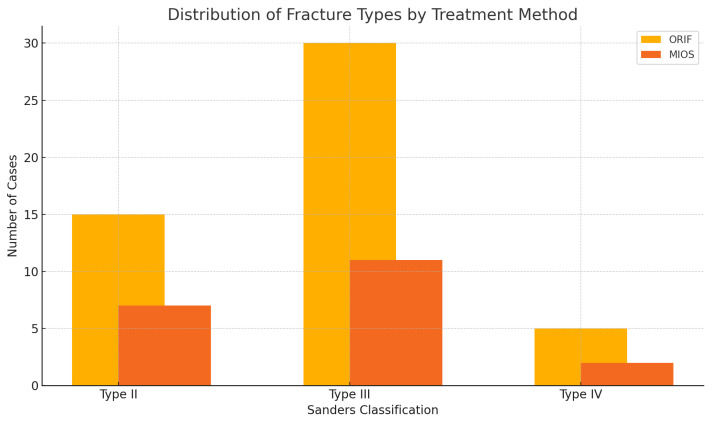
Distribution of fracture types by treatment method. The figure shows the number of cases for each Sanders classification type in the ORIF and MIOS groups. ORIF group included 15 type II, 30 type III, and 5 type IV fractures. MIOS group included 7 type II, 11 type III, and 2 type IV fractures.

A chi-square analysis was conducted to examine the correlation between Sanders classification and the choice of treatment (ORIF vs. MIOS). The results indicated no significant correlation between the complexity of the fracture and the type of treatment chosen (χ^2^ = 0.175, *p* = 0.916). Additionally, a Cochran–Armitage test for trend was performed, confirming that there was no significant trend in the choice of treatment based on fracture complexity (statistic = 0.048, *p* = 0.826). Patient distributions for these treatment techniques are shown in Figure 1.

### 3.4. Radiographic Outcomes

The radiographic assessment showed that the mean preoperative Böhler’s angle was 5° ± 4.2° for the ORIF group and 9° ± 5.1° for the MIOS group (*p* = 0.012). Postoperatively, the mean Böhler’s angle improved to 30° ± 6.3° in the ORIF group and 24° ± 7.4° in the MIOS group (*p* < 0.001). The mean preoperative Gissane’s angle was 120° ± 9.2° in the ORIF group and 118° ± 8.7° in the MIOS group (*p* = 0.298). Postoperatively, the Gissane’s angle was restored to 130° ± 7.8° in the ORIF group and 128° ± 6.9° in the MIOS group (*p* = 0.215).

### 3.5. Functional Outcomes

Functional outcomes were assessed using the Maryland Foot Score (MFS) and the Creighton-Nebraska Health Foundation Assessment Scale (CNHFAS). The mean MFS score was 85 ± 10 in the ORIF group and 80 ± 12 in the MIOS group (*p* = 0.124). The mean CNHFAS score was 87 ± 9 in the ORIF group and 82 ± 11 in the MIOS group (*p* = 0.088). There were no statistically significant differences in the functional outcomes between the two groups.

### 3.6. Complications

The overall complication rates did not significantly differ between the ORIF and MIOS groups. Immediate complications, such as traumatic or hemorrhagic shock, were rare and occurred in less than 5% of cases in both groups. Early complications, including thromboembolism, wound dehiscence or infection, and compartment syndrome, were reported in 10% of the ORIF group and 15% of the MIOS group (*p* = 0.231). Late complications, such as chronic pain and swelling, tendinitis, joint stiffness, and arthritis, were observed in 30% of the ORIF group and 25% of the MIOS group (*p* = 0.412). The chi-square test analysis of postoperative complications showed no significant differences between the ORIF and MIOS groups for tendinitis, chronic pain and swelling, algodystrophy, and surgical wound infections (*p* > 0.20 for all).

### 3.7. Reintervention Rates

**Figure 2:**
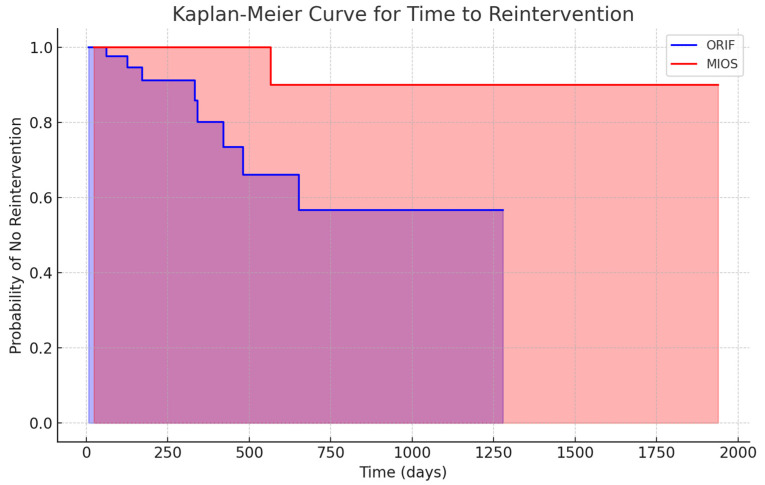
Kaplan–Meier curve for time to reintervention between ORIF and MIOS groups.

Reintervention rates were higher in the ORIF group (18%) compared to the MIOS group (5%) (*p* = 0.034). A significant reduction in the risk of reintervention was observed in patients with high anesthetic risks, polytraumatized individuals, and those with comorbidities who underwent MIOS. The Kaplan–Meier survival analysis illustrated in Figure 2 shows a significantly longer time to reintervention for patients treated with MIOS compared to ORIF (*p* = 0.029).

### 3.8. Quality of Life

**Figure 3:**
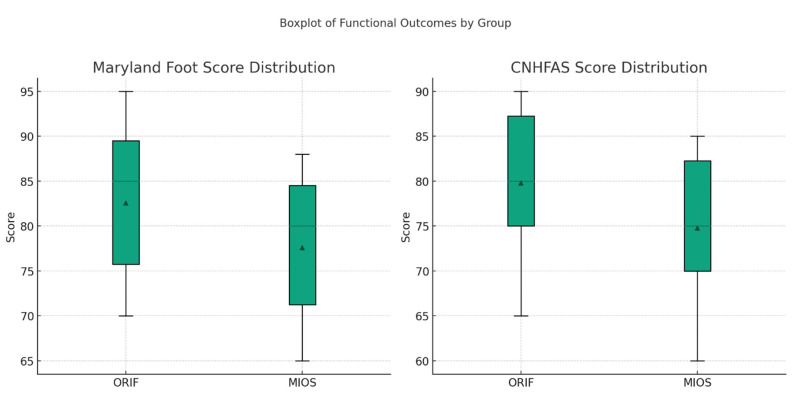
The distribution of Maryland Foot Score and CNHFAS across ORIF and MIOS groups.

The quality of life, as measured by the MFS and CNHFAS, showed no significant differences between the ORIF and MIOS groups. Both groups reported high levels of satisfaction with the surgical outcomes, with 85% of MIOS patients and 80% of ORIF patients reporting good to excellent results (*p* = 0.502). Using the Shapiro–Wilk test, we determined that the MFS and the CNHFAS distributions in both ORIF and MIOS groups were normal (*p* > 0.05). Later analyses through independent *t*-tests showed no noteworthy differences in these functional outcomes between the groups (*p* > 0.20). This indicates that the two treatments had comparable efficiency regarding functional recovery (Figure 3).

### 3.9. Summary of Results

Table 1 provides a comparative overview of treatment outcomes between ORIF and MIOS for calcaneal fractures, including patient demographics, radiographic outcomes, functional outcomes, complication rates, reintervention rates, and quality-of-life assessments.

## 4. Discussion

The treatment paradigm for displaced intra-articular calcaneal fractures (DIACF) has significantly evolved over recent decades. The primary focus has shifted from achieving precise anatomical reduction of the subtalar joint to restoring the three-dimensional height and correct alignment of the calcaneal body in axial and coronal planes. Modern strategies now emphasize postoperative care and its broader impact on patients’ functional reintegration and quality of life, extending beyond traditional measures like range of motion (ROM) and chronic pain to consider complications in soft tissue healing [2,11].

Interest in minimally invasive techniques such as percutaneous reduction and external fixation is growing. These techniques are especially useful for treating high-energy fractures accompanied by severe soft tissue damage, as well as for patients with added risk factors such as old age, immunosuppression, diabetes, smoking, coagulopathy, and high anesthetic risk [12,13].

While there is a lack of randomized clinical trials comparing ORIF to external limb alignment and MIOS with external fixation, meta-analyses indicate that there are no substantial outcome differences between these treatments. Although ORIF often results in better anatomical restoration of the Böhler’s angle, it matches the functional results of other methods. However, it is linked with a higher occurrence of postoperative complications such as surgical wound dehiscence, infections, neurovascular injuries, and early osteoarthritis. Conversely, MIOS with external fixation tends to yield fewer complications, which are also less severe. These include chronic pain, occasional infections at the screw insertion sites, inadequate reduction, and postoperative osteoarthritis [1,8,9,13,14].

### 4.1. Correlation between Sanders Classification and Treatment Choice

Our chi-square analysis indicated no significant correlation between the Sanders classification and the choice of treatment (χ^2^ = 0.175, *p* = 0.916). More complex fractures such as Sanders types III and IV were not significantly more frequently treated with ORIF compared to less severe fractures such as Sanders type II. Additionally, the Cochran–Armitage test for trend showed no significant trend in the choice of treatment based on fracture complexity (statistic = 0.048, *p* = 0.826). This suggests that the choice between ORIF and MIOS was not influenced by the severity of the fractures as classified by Sanders. These findings contrast with previous studies, which reported that surgical approach was influenced by fracture type in accordance with the Sanders classification [5,10]. Our results indicate that other factors may play a more significant role in the decision-making process for choosing the surgical method [5,10].

### 4.2. Clinical Implications of ORIF and MIOS

The choice of ORIF over MIOS, despite similar results in terms of MFS and CNHFAS, can be justified by the superior anatomical restoration achieved with ORIF, as evidenced by the significant improvement in the Böhler’s angle. The restoration of the Böhler’s angle is crucial for maintaining the biomechanics of the heel and preventing long-term complications such as post-traumatic arthritis. ORIF provides better stability and alignment, which are particularly important for high-demand patients who require optimal anatomical outcomes for their functional recovery and long-term activity levels. Additionally, ORIF may be preferable in cases where the precise reduction of complex fractures is necessary to ensure proper healing and function. The Kaplan–Meier survival analysis indicated a significantly longer time to reintervention for MIOS compared to ORIF, particularly in high-risk patients. This suggests that MIOS may be more suitable for patients with high anesthetic risks, polytrauma, or comorbidities due to its less invasive nature and lower immediate postoperative complication rates.

### 4.3. Complications and Quality of Life

The overall complication rates did not significantly differ between the ORIF and MIOS groups. However, specific complications varied, with ORIF patients experiencing higher rates of tendinitis and joint stiffness, while MIOS patients had a higher incidence of osteoarthritis and chronic pain. Quality-of-life assessments using the Maryland Foot Score (MFS) and Creighton-Nebraska Health Foundation Assessment Scale (CNHFAS) showed no significant differences between the groups, indicating comparable long-term functional outcomes.

The chi-square test analysis of postoperative complications highlighted no substantial differences between the ORIF and MIOS groups in relation to tendinitis, chronic pain and swelling, algodystrophy, and surgical wound infections (*p* > 0.20 for all). Additionally, the Kaplan–Meier survival analysis illustrated in Figure 2 shows a significantly longer time to reintervention for patients treated with MIOS compared to ORIF (*p* = 0.029).

### 4.4. Recommendations for Clinical Practice

Based on our findings, we recommend that the choice between ORIF and MIOS should be tailored to the individual patient’s health status, fracture severity, and functional requirements. ORIF is preferable for patients with high functional demands due to its superior anatomical outcomes, whereas MIOS is more advantageous for high-risk patients as it minimizes the need for reinterventions and has a lower risk of immediate complications.

### 4.5. Future Research Directions

Further research with larger patient cohorts and extended follow-up periods is essential to validate our findings and develop precise, evidence-based guidelines for managing calcaneal fractures. Randomized controlled trials are particularly needed to eliminate selection bias and provide high-quality comparative data on the efficacy and safety of ORIF versus MIOS.

### 4.6. Selection Bias and Study Limitations

One major limitation of this study is the selection bias introduced by allowing the choice of surgical technique (ORIF vs. MIOS) to be determined by the surgeon’s preference rather than predefined criteria. This subjective decision-making process inherently limits the generalizability of our findings. Consequently, the results should be interpreted with caution. Future studies should aim to use randomized controlled trials to eliminate such biases and provide more robust comparative data. We also acknowledge the inclusion of patients over 65 years of age, which may have introduced a bias due to the increased difficulty in healing and higher risk of complications associated with older age. This factor should be considered when interpreting the study results, and future studies should consider stratifying patients by age to better understand its impact.

Another significant bias in our study is the unequal gender distribution between the ORIF and MIOS groups. The ORIF group had a higher proportion of male patients compared to the MIOS group. This discrepancy could potentially influence the outcomes, as gender differences in bone healing, muscle strength, and overall physical condition might affect recovery and complication rates. Specifically, males typically have greater bone density and muscle mass, which could contribute to differences in recovery trajectories and functional outcomes. To mitigate this bias in future research, studies should aim for a more balanced gender distribution or apply statistical methods to adjust for gender differences. Additionally, conducting subgroup analyses based on gender could provide deeper insights into how each treatment modality affects males and females differently.

## 5. Conclusions

Both ORIF and MIOS are effective in managing calcaneal fractures, with each technique offering distinct advantages based on patient-specific factors. The findings from this study provide valuable insights that can inform clinical decision-making and highlight the importance of individualized treatment plans.

## Figures and Tables

**Table 1:** Comparative overview of treatment outcomes between ORIF and MIOS for calcaneal fractures.

Parameter	ORIF (*n* = 50)	MIOS (*n* = 20)	*p*-Value
Mean Age (years)	46.6 ± 12.4	53.5 ± 11.2	0.032
BMI (kg/m^2^)	25.8 ± 3.4	26.3 ± 3.7	0.452
Gender (M/F)	28/12	14/6	0.801
Mean Waiting Time for Surgery (days)	9 ± 3	3 ± 2	<0.001
Preoperative Böhler’s Angle (°)	5 ± 4.2	9 ± 5.1	0.012
Postoperative Böhler’s Angle (°)	30 ± 6.3	24 ± 7.4	<0.001
Mean MFS Score	85 ± 10	80 ± 12	0.124
Mean CNHFAS Score	87 ± 9	82 ± 11	0.088
Early Complications (%)	10	15	0.231
Late Complications (%)	30	25	0.412
Reintervention Rates (%)	18	5	0.034
Quality of Life (Good to Excellent)	80%	85%	0.502

## Data Availability

Photos of the surgical techniques are available upon request.
